# Pinocembrin mitigates depressive-like behaviors induced by chronic unpredictable mild stress through ameliorating neuroinflammation and apoptosis

**DOI:** 10.1186/s10020-020-00179-x

**Published:** 2020-05-27

**Authors:** Wei Wang, Lili Zheng, Lijun Xu, Jianglong Tu, Xunhu Gu

**Affiliations:** 1 Department of Neurology, The Second Affiliated Hospital of Nanchang University, No.1 Minde Road, Nanchang City, Jiangxi Province China; 2 Department of Pharmacy, Jiangxi Maternal and Child Health Hospital, Nanchang City, 330006 Jiangxi Province China

**Keywords:** Pinocembrin, Chronic unpredictable mild stress, Neuroinflammation, Apoptosis, Oxidative stress

## Abstract

**Background:**

The majority of patients with chronic fatigue have a risk of comorbidity with depression. Pinocembrin (PB) is a kind of flavonoid molecule isolated from honey and propolis and has antimicrobial, anti-inflammatory, antioxidant, and anticancer function. The purpose of the current study was to determine the possible function of PB on treatment of depression.

**Methods:**

A chronic unpredictable mild stress (CUMS) mouse model was established to mimic the depressive-like behaviors in vivo. The depressive-like behaviors of CUMS mice were measured by sucrose preference test (SPT), open field test (OFT), forced swim test (FST) and tail suspension test (TST). The concentration of reactive oxygen species (ROS), malondialdehyde (MDA) and the activity or superoxide dismutase (SOD) were detected by commercial kit. The inflammatory factor including interleukin (IL)-1β, tumor necrosis factor (TNF)-α, IL-10 and transforming growth factor (TGF)-β were examined.

**Results:**

We found that PB alleviated the decreasing of sucrose preference and body weight. CUMS mice significantly increased the immobility time but decreased latency to abandon in FST, increased the immobility time in TST, and reduced crossing score and rearing score in OFT, whereas these changes were reversed by PB treatment. More importantly, PB decreased the concentration of ROS and MDA, but increased the SOD activity, suggesting that it could protected against oxidative stress in CUMS mice. Interestingly, PB inhibited cell apoptosis and regulated inflammatory factors expression in hippocampus of CUMS mice. Moreover, PB activated Nrf2/HO-1 signal pathway but inhibited the phosphorylation of NF-kB.

**Conclusions:**

In conclusion, PB mitigated CUMS-induced depressive-like behaviors through ameliorating neuroinflammation and apoptosis.

**Trial registration:**

Not Applicable.

## Background

Depression is a severe and recurrent disease, which is characterized by persistent depressed mood and impaired cognitive functions, even leads to suicide or self-harm (Coleman et al. 2019; Butter et al. 2019). More patients have been affected in the world, thus it becomes a serious personal pain and social problem. Currently, more than 20 different antidepressants are used to treat depression, however, there is still a large of patients suffering the pains which are brought from depression (Kessler and Bromet 2013). The main reason of the poor effect of antidepressant treatment is the ambiguity of the pathophysiology of depression and the molecular mechanism of antidepressant treatment (Riddle et al. 2017; Peng 2018). Therefore, further studies about the pathogenesis of depression and therapeutic strategies are needed.

The brain is susceptible to oxidative stress because of its high iron content, high oxygen consumption, low antioxidant capacity and peroxidation of fatty acids (Madrigal et al. 2006; Salim 2017). Therefore, cerebral oxidative stress is an important mechanism of brain dysfunction, especially depression and anxiety (Tell and Gustincich 2009). In a previous study, cell apoptosis is considered as a mechanism for promoting stress-related mood disorders (Lee et al. 2014). Cell death occurs in specific groups of neurons, which are caused by chronic stress. In the circumstances, antidepressants have been shown to improve repetitive stress-induced cognitive impairment (Kwon et al. 2013). In clinical patients, the release of pro-inflammatory cytokines, especially interleukin-1 cytokines (IL-1) and tumor necrosis factor (TNF-), is higher in depressed patients compared with normal patients, indicating that inflammation plays an vital role in the pathophysiology of depression (Al-Hakeim et al. 2015; Eyre et al. 2016; Hannestad et al. 2011). In addition, antidepressant treatment reduces serum inflammatory cytokines (Yamawaki et al. 2018). Higher expression of pro-inflammatory cytokines have been identified in depressed animal models (Jiang et al. 2017a). Therefore, these findings suggest that the anti-inflammatory and anti-apoptotic functions play vital roles in depression treatment.

Natural products are valuable and novel resources for drug development, such as propolis. Pinocembrin (5,7-dihydroxyflavanone, molecular formular: C_15_H_12_O_4_, molecular weight: 256.25 g/mol), is a kind of flavonoid, which is isolated from propolis and honey (Rasul et al. 2013). The PB showed antioxidant and anti-inflammatory abilities and neuroprotective functions in vivo and in vitro (Reddy et al. 2013; Lan et al. 2016). PB alleviates inflammation, oxidative interference, and apoptosis in the hippocampus of the brain ischemia-reperfusion rat model (Saad et al. 2015). However, it has not been reported whether it can alleviate depression-like behaviors with the mechanism of inflammation and apoptosis. The purpose of our study was to study the regulation of PB on depression in a CUMS-induced depression mouse model.

## Methods

This study was obeyed the Guide for the Care and Use of Laboratory Animals and the protocol was approved by the Ethics Committee of The Second Affiliated Hospital of Nanchang University.

### Animals and treatment

**Fig. 1:**
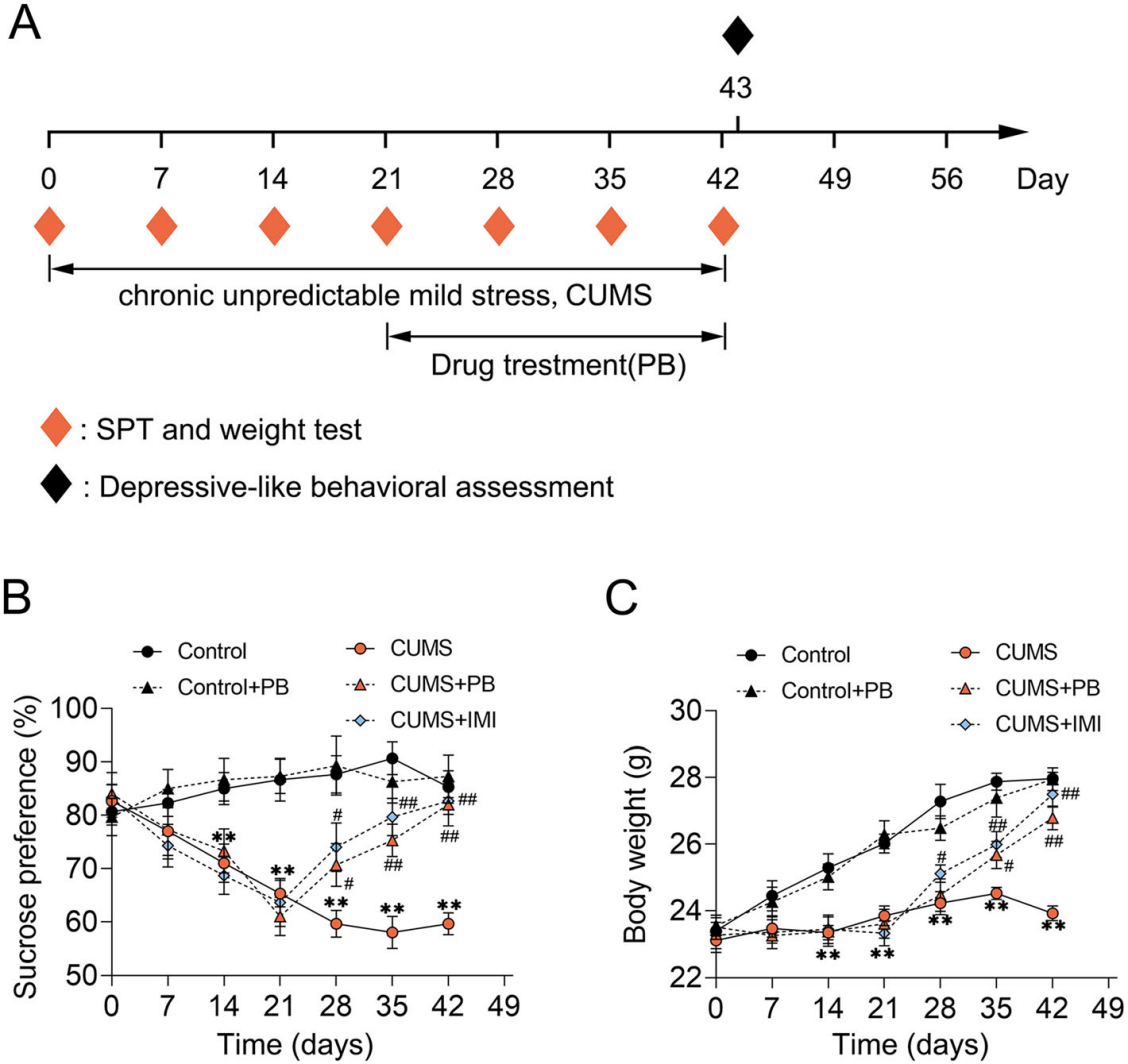
PB alleviated sucrose preference and body weight in CUMS mice (**A**) Experimental schedule. A total of 40 C57BL/6 J mice randomly divided into 5 groups: (a) Control; (b) Control+PB; (c) CUMS; (d) CUMS+PB; (e) CUMS+IMI. Meanwhile, the IMI treatment was served as a positive control, and IMI was administrated every day during the UCSM experiments by oral gavage. The unpredictable chronic mild stress (UCMS) protocols and PB treatment lasted 6 weeks and 3 weeks, respectively. Before the UCMS procedure, the sucrose preference and body weight were measured and lasted for once a week. Sucrose preference was measured in UCMS weekly. The depressive-like behavioral assessment was assessed at the end of the experiments. The sucrose preference (**B**) and body weight (**C**) were detected once a week. Eight mice per group. **p < 0.01 vs. Control. #*p* < 0.05, ##*p* < 0.01 vs. CUMS

Total of 40 male C57BL/6 J mice (six-week-old) were purchased from Huafukang Company. Every mouse was fed adaptively under a normal 12 h light/dark cycle at 23 ± 2 °C (humidity 45%–55%) for 2 weeks before experiments began. During the study, the mice were given food and water every day. The mice were randomly divided into five groups (*n* = 8 per group):

- Control;
- Control+ 10 mg/kg PB;
- Chronic unpredictable mild stress (CUMS);
- CUMS+ 10 mg/kg PB;
- CUMS+ 10 mL/kg imipramine hydrochloride (IMI);

The CUMS experiments were performed for 6 weeks. At the 4th week, the PB was administrated once a day for 3 weeks by oral gavage. The IMI treatment was served as a positive control, the IMI were administrated every day during the UCSM experiments by oral gavage. The UCSM and SPT, depressive-like behavioral assessment were carried out in the order presented in Fig. 1A.

### Chronic unpredictable mild stress (CUMS)

To explore the effects of PB on depression-like behaviors in animals, the CUMS-induced mouse model was used in this study. The control group did not receive any of the chronic unpredictable mild stresses experiment, whereas CUMS-induced mice received single housed and subjected to one of the following stressors once every day in a randomized fashion: (I) lake of food for 24 h, (II) lake of water for 24 h (III) lighting for 24 h, (IV) cage tilt (45°), (V) the damp environment (per 100 g padded add 200 ml of water), (VI) exposed to the external environment, (VII) the light light/dark cycle, (VIII) alternately suspension for 10 min, (IX) exposed to an empty bottle, (X) clip tail (1 min, 1 cm in from the tail to start), (XI) oscillation for 5 min, (XII) white noise. Mice were subjected to different stressors daily for 42 days in an unpredictable way. Finally, the mice were sacrificed at the 43th day. The mice anesthetized with sodium pentobarbital (100 mg/kg) and fentanyl (0.05 mg/kg). The brains were quickly removed, washed with phosphatic buffer solution (PBS), and post-fixed in 4% paraformaldehyde overnight at 4 °C for further experiments.

### Sucrose preference test (SPT)

Anhedonia is the inability to experience pleasure from rewarding or enjoyable activities, which is a core symptom of depression in humans, and can be assessed by SPT assay (Liu et al. 2018). In this experiment, weight was weighed every 7 days for 6 weeks. In addition, SPT was administered every 7 days before and after induction in CUMS. First, the mice were placed in separate cages, each adapted to a 1% sucrose solution (w/v). After 24 h of exposure to two bottles of sucrose solution, a bottle of 1% sucrose solution in each cage was replaced with a bottle of water and exposed for another 24 h. Then, all mice were deprived of water and food for 24 h, and SPT was administered for 12 h. During this time, the mice were free to use two bottles, one contained a 1% sucrose solution and the other was only water. The location of the bottle was changed during the 6-h test to avoid possible side effects. The sucrose preference was calculated as follows: SPT (%) = ((sucrose intake (g)/(sucrose intake (g) + water intake(g))) × 100.

### Forced swim test (FST)

The FST is a model of depressive-like behavior (Yankelevitch-Yahav et al. 2015). To allow adaptation to the environment, all mice were taken by a 6 min FST. The mice in each group were placed in a water container 25 cm high and 10 cm in diameter (10 cm deep; 23 ± 2 °C). The total 6-min process included the first 2-min unmeasured swim and the subsequent 4-min swim. The immobility time of 4 min after swimming was recorded by the software tracking analysis system (Xinsoft Information Technology, China). The latency to abandon is thought to be the time which ranged from the time that the animal was introduced into the pool to the time it first stopped completely (at least 2 s).

### Tail suspension test (TST)

The TST assay is a widely used models for assessing antidepressant-like activity in animal models (Cryan et al. 2005). In this study, the TST assay was performed as a previous reference described (Jiang et al. 2017b). Each mouse was hung 50 cm away from the ground for 6 min, with its tail attached to the hook (about 2 cm from the end). Calculate the immobility time for the last 4 min. The mice are considered to be motionless only when they are completely motionless.

### Open field test (OFT)

The OFT experiment was a test of anxiety-related behavior and locomotion and performed as described previously (Wu et al. 2019; Belzung and Griebel 2001). In short, the instrument is divided into 12 equal squares. The rats were then placed in the middle of the field and allowed to explore their surroundings Finally, the numbers of square crossings, rearing and grooming scores within 5 min of free activity were recorded.

### Measurement of MDA and SOD

The hippocampus from mice were homogenized (10% w/v) with PBS and centrifuged for collecting supernatants. The levels of MDA and the activity of SOD in the supernatant were respectively measured by MDA Assay Kit (Abnova, USA) and SOD Assay Kit (Abnova, USA), according to the manufacturer’s instructions.

### Determination of ROS level

The levels of ROS in hippocampus were evaluated by using 2, 7-dichlorofluorescindiacetate (DCFDA) Kit (Sigma, China). Briefly, hippocampus homogenate was washed with PBS, mixed with DCFDA (dissolved with methanol) and then incubated in water for 15 min at 37 °C. After that, the samples were stained by DAPI. ROS images were measured using a DCFH-DA fluorescence probe under a fluorescence microscope with a magnification of 400×. Fluorescence intensity was calculated using a fluorometer at 488 nm excitation wavelength and 525 nm emission wavelength (Hemmati et al. 2018).

### Terminal-deoxynucleoitidyl Transferase mediated Nick end labeling (TUNEL) assay

The cell apoptosis was detected by FragEL™ DNA Fragmentation Dectection Kit (Merck-Calbiochem, USA). The hippocampus from mice were embedded in paraffin, and cut into 5-μm slices. After deparaffinization, the tissue sections were incubated with proteinase K for 25 min and incubated with H_2_O_2_ in methanol (8 min). Next, the sections were placed in the TUNEL reaction mixture and incubated in darkness for 60 min. After washing by PBS, the sections were incubated with streptavidin-horseradish peroxidase (HRP) conjugate, and DAB was served as chromogen substrate. The nuclear staining of cell in intense and brown was TUNEL-positive.

### Immunohistochemistry assay

The hippocampus from mice after deparaffinization were incubated in citrate buffer solution, and then were blocked with 10% goat serum. After that, slides incubated with the Anti-Cleaved Caspase-3 antibody (dilution:1:300; Abcam, USA) at 4 °C overnight. Then, the sections were stained with DAB chromogen substrate in the dark. Finally, the images were recorded by a microscope.

### Measurement of TNF-α, IL-1β, TGF-β and IL-10

The concentrations of TNF-α, IL-1β, TGF-β and IL-10 in hippocampus from mice were measured by Mouse IL-1β ELISA Kits (MULTI SCIENCES, China), Mouse TNF-α ELISA Kits (MULTI SCIENCES, China), Mouse IL-10 ELISA Kits (MULTI SCIENCES, China) or Mouse TGF-β ELISA Kits (MULTI SCIENCES, China).

### Quantitative real time polymerase chain reaction (qRT-PCR)

Total RNA was extracted from hippocampus in mice using TRIzol reagent (Takara, Japan). And then the cDNA was synthesized by Prime-Script™ RT regent Kits (Takara, Japan). The mRNA expression levels were measured using qRT-PCR by SYBR Premix Ex Taq (Takara, Japan). The critical threshold cycle (Ct) value was determined and the relative quantification data were calculated with the 2^-ΔΔCt^ method, the GAPDH was served as a reference. The primers were as follows: IL-1β-forward: 5′-GAGTCTGCACAGTTCCCCAA-3′, IL-1β-reverse: 5′-TGTCCCGACCATTGCTGTTT-3′; TNF-α-forward: 5′-CGTC AGCCGATTTGCCATTT-3′, TNF-α-reverse: 5′-CTCCCTCAGGGGTGTCCTTA-3′; TGF-β-forward: 5′-GAACTCGCTTTGTCTCCA-3′, TGF-β-reverse: 5′-TACAGTCGCAGTATAACCTCA-3′; IL-10-forward: 5′-TCTCCGAGATGCCTTCAGCAGA-3′, IL-10-reverse: 5′-TCAGACAAGGCTTGGCAACCCA-3′; GAPDH-forward: 5′-ATGGGGAAGGTGAAGGT-3′, GAPDH-reverse: 5′-AAGCTTCCCGTTCTCAG-3′.

### Western blot

The protein from the tissues of mice were lysed with RIPA lysis buffer (Beyotime, China). Then, the protein concentrations were detected by a BCA protein kit (Beyotime, China). Next, equal proteins from different groups were separated by SDS-poluacrylamide gel electrophoresis, transferred onto a PVDF membrane. After washed by TBST buffer for three times, the membrane was incubated with first antibodies: Nrf2 antibody (dilution: 1:400; Abcam, USA), HO-1 antibody (dilution: 1:400; Abcam, USA), Bcl-2 antibody (dilution: 1:800; Proteintech, USA), Bax antibody (dilution: 1:800; Proteintech, USA), p-NF-kB antibody (dilution: 1:400; Abcam, USA), NF-kB antibody (dilution: 1:400; Abcam, USA), GAPDH antibody (dilution: 1:10000; Proteintech, USA) overnight at 4 °C, followed by incubation with the appropriate secondary antibodies (dilution: 1:5000; Beyotime, China) for 2 h. The GAPDH was served as a loading control.

### Data analysis

All Data were expressed as mean ± standard deviation (SD) and analyzed with were analyzed by one-way analysis of variance (ANOVA) or two-way ANOVA by GraphPad Prism 7.0. *P* < 0.05 was considered significant.

## Results

### PB alleviated sucrose preference and body weight in CUMS mice

The UCSM and SPT, depressive-like behavioral assessment were presented in Fig. 1A. Firstly, the CUMS treatment markedly reduced the sucrose preference and body weight than control mice. Nevertheless, the PB or IMI treatment could alleviate the decreasing of sucrose preference and body weight (Fig. 1B and C). Thus, we could conclude that PB alleviated sucrose preference and body weight in CUMS mice.

### PB alleviated depressive-like behaviors of CUMS mice

**Fig. 2:**
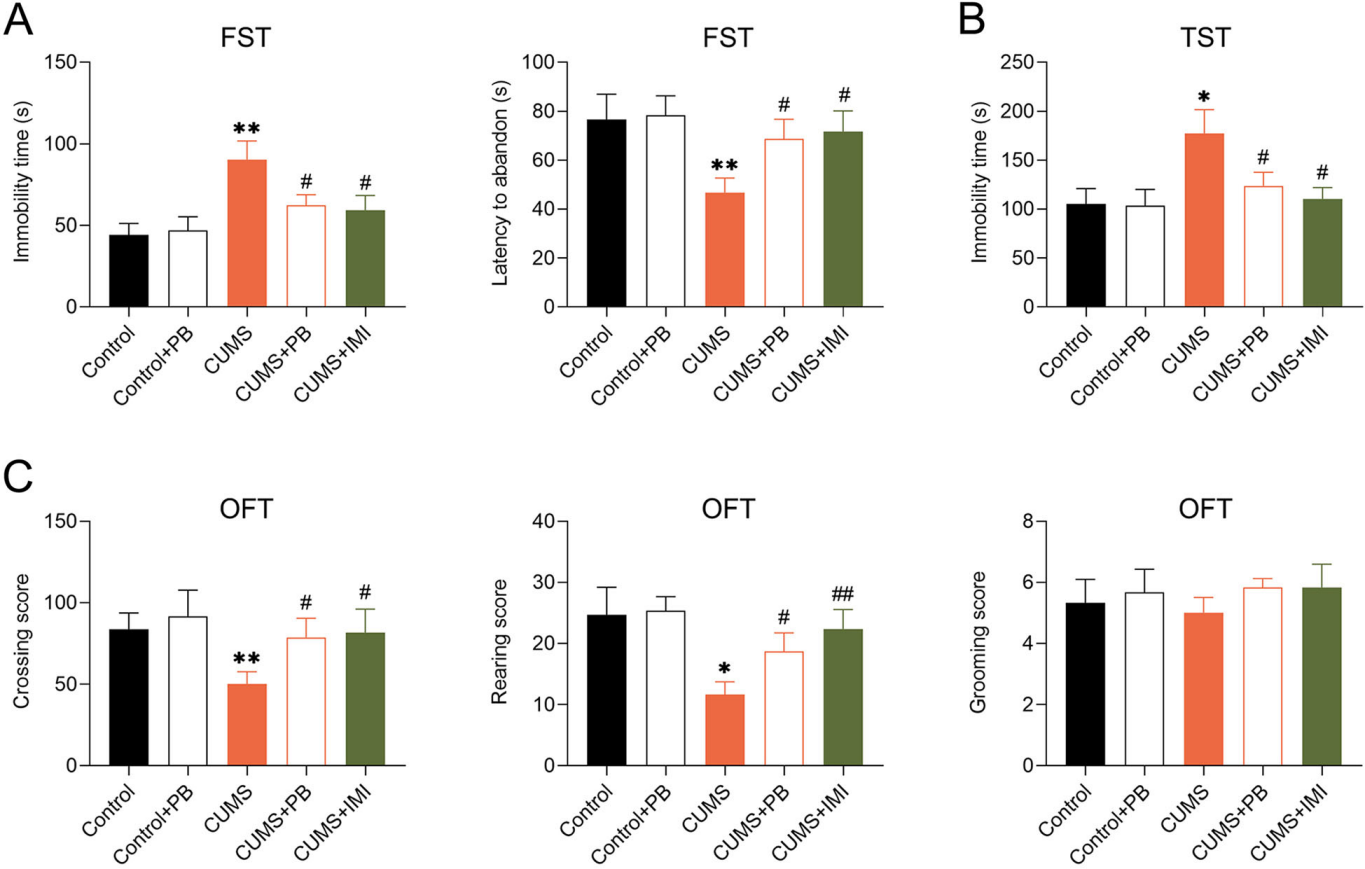
PB alleviated depressive-like behaviors of CUMS mice **a** PB decreased the immobility time but increased latency to abandon of CUMS mice in the forced swimming test (FST). **b** PB reduced the immobility time of CUMS mice in the tail suspension test (TST). **c** PB promoted crossing score and rearing score of CUMS mice in the open field test (OFT), with no change in grooming score. Eight mice per group. **p < 0.01 vs. Control. #*p* < 0.05, ##*p* < 0.01 vs. CUMS

To clarify the function of PB on CUMS mice, the depressive-like behaviors was detected by Depressive-like behavioral assessment. Six-week CUMS experiments markedly increased the immobility time but decreased latency to abandon in FST compared to control mice. Treatment with PB or IMI significantly decreased immobility time but increased latency to abandon in FST compared to that of the CUMS (Fig. 2a). Moreover, compared to control mice, PB observably increased the immobility time in TST, as well as IMI (Fig. 2b). The PB or IMI treatment significantly increased crossing score and rearing score compared to CUMS mice in OFT. Nevertheless, there was no significant difference among the five groups in grooming score (Fig. 2c). These findings indicated that PB alleviated depressive-like behaviors of CUMS mice.

### PB protected against oxidative stress in hippocampus of CUMS mice

**Fig. 3:**
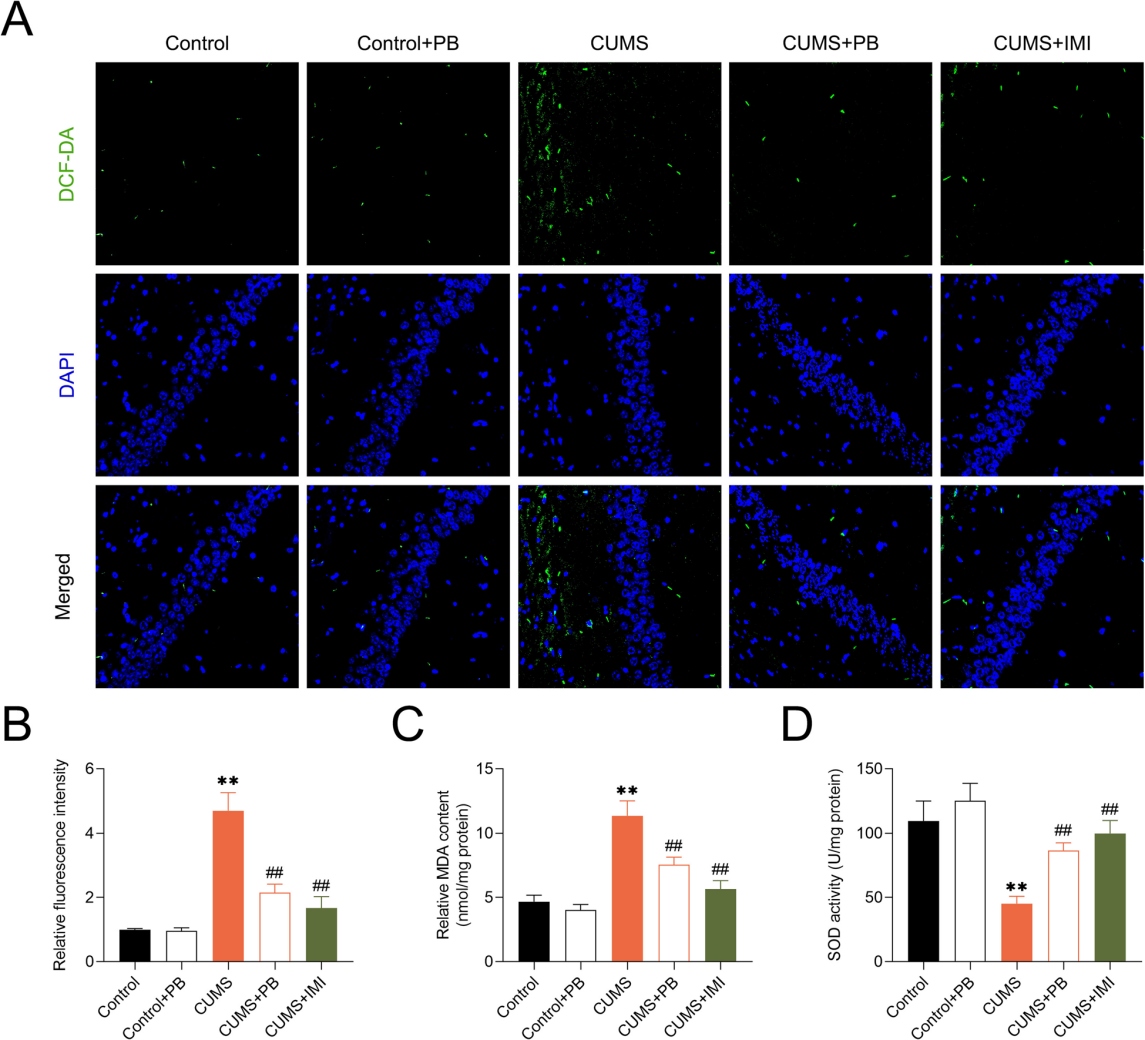
PB protected against oxidative stress in hippocampus of CUMS mice **a** PB inhibited ROS production, which was measured using the DCFH-DA fluorescent probe under a fluorescence microscope at a magnification of × 400. **b** The fluorescence intensity was calculated and was correlated with the ROS level. **c** The concentration of MDA was measured, suggesting that PB decreased MDA concentration in CUMS mice. **d** The activity of SOD was measured, indicating that PB promoted SOD activity in CUMS mice. **p < 0.01 vs. Control. ##*p* < 0.01 vs. CUMS

To further investigate the role of PB in the oxidative stress, the levels of ROS, MDA and SOD in the hippocampus of CUMS-treated mice were measured. ROS level was significantly promoted in the hippocampus of CUMS mice compared to control mice. PB or IMI treatment markedly inhibited the ROS production (Fig. 3A and B). The concentration of MDA (Fig. 3c) was significantly increased but the activity of SOD (Fig. 3d) were markedly decreased in CUMS-induced mice. Nevertheless, treatment with PB or IMI decreased the concentration of MDA but increased the SOD activity in CUMS-induced mice, indicating that PB protected against CUMS-induced hippocampal oxidative stress.

### PB inhibited cell apoptosis in hippocampus from CUMS mice

**Fig. 4:**
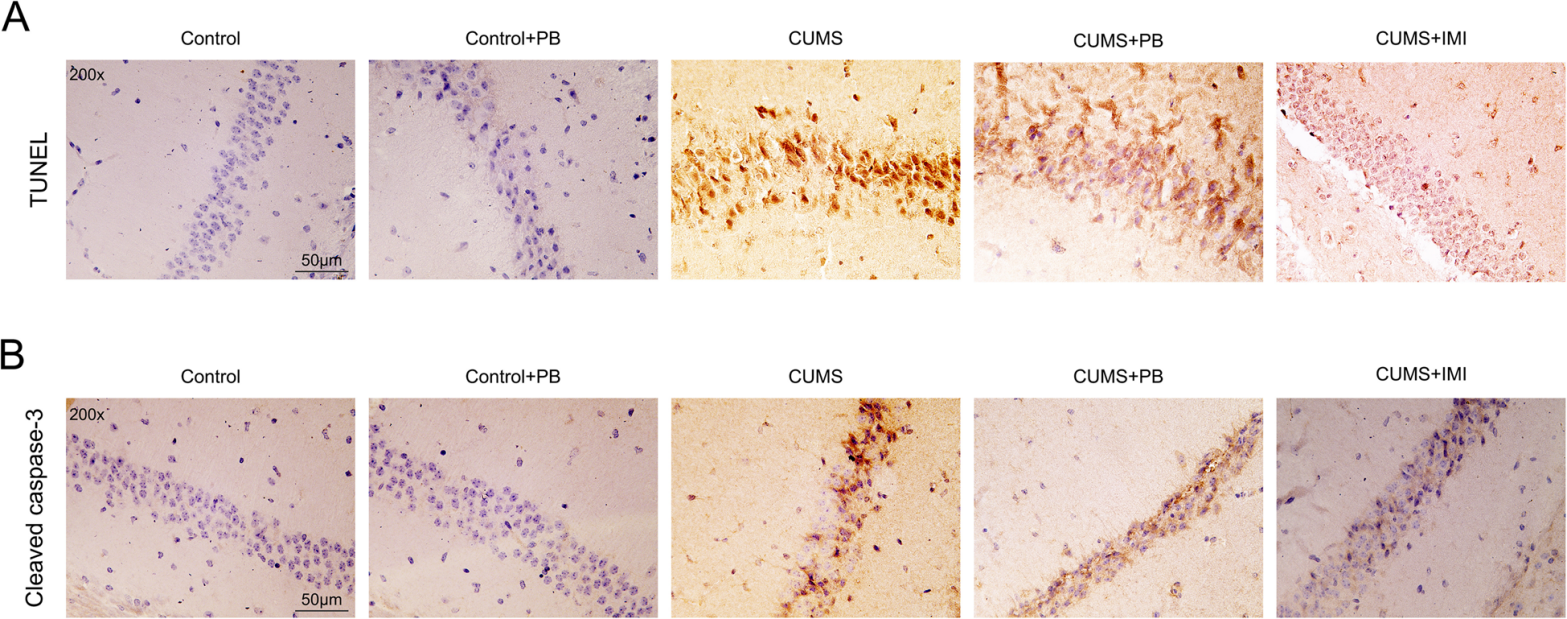
PB inhibited the cell apoptosis in hippocampus from CUMS mice The cell apoptosis in hippocampus of CUMS mice was determined by TUNEL assay and IHC assay of cleaved caspase-3 (200×), bar = 50 μm

The apoptotic neurons in hippocampus was recognized by TUNEL staining and IHC assay in the hippocampus from mice. In the Control and Control+PB mice, TUNEL-positive cells or cleaved caspase-3-positive cells were rare or absent in hippocampus (Fig. 4a). Nevertheless, the TUNEL-positive cells or cleaved caspase-3-positive cells were present in hippocampus from CUMS mice. Interestingly, after the PB or IMI treatment, the TUNEL-positive cells or cleaved caspase-3-positive cells were redyced in hippocampus from CUMS mice (Fig. 4b). These findings proved that PB inhibited the cell apoptosis in hippocampus from CUMS mice.

### PB regulated inflammatory factors expression in CUMS mice

**Fig. 5:**
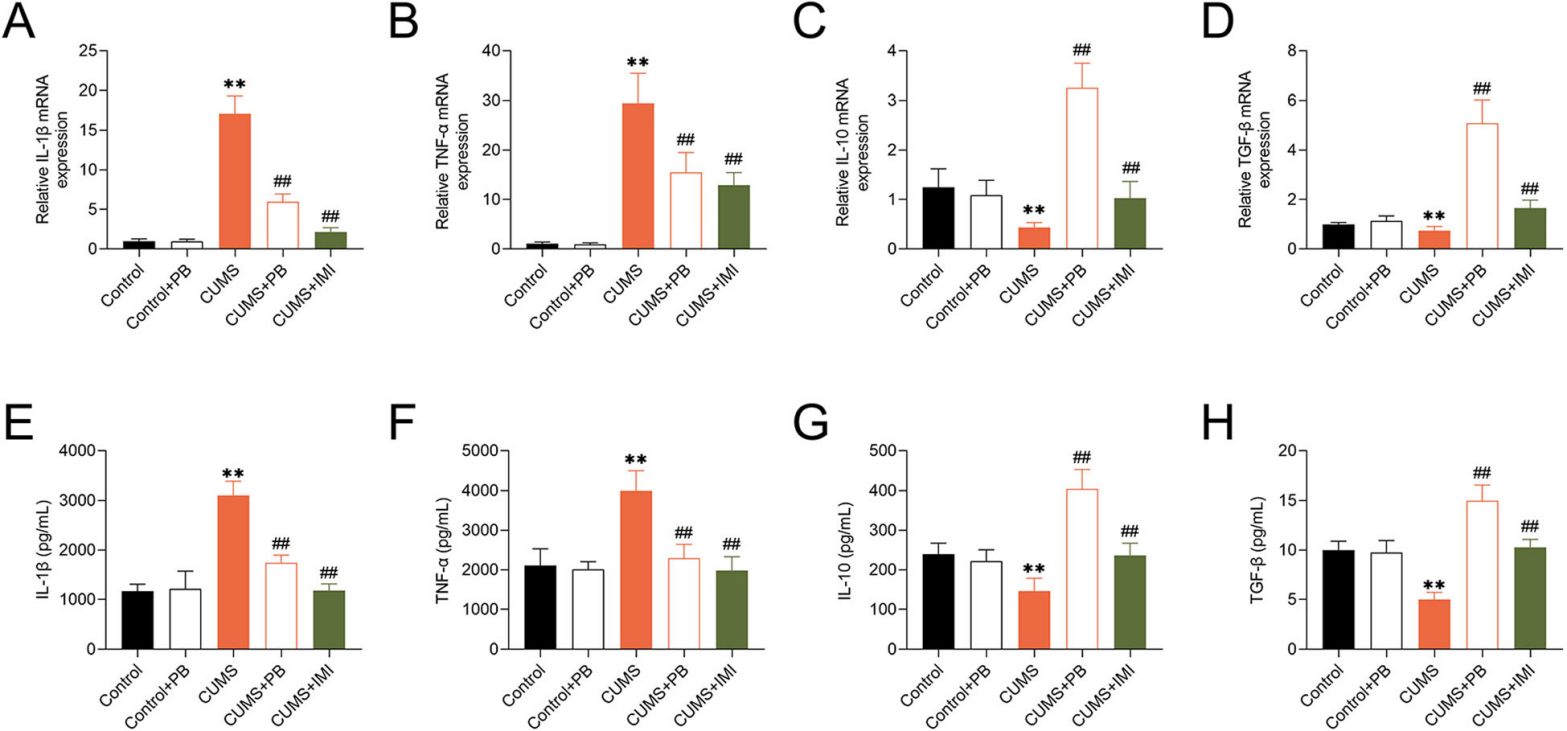
PB regulated inflammatory factors expression in CUMS mice B The expression of IL-1β, TNF-α, IL-10 and TGF-β in mice were determined by qRT-PCR. **b** The concentrations of IL-1β, TNF-α, IL-10 and TGF-β in hippocampus of mice were detected by ELISA assay. **p < 0.01 vs. Control. ##*p* < 0.01 vs. CUMS

Compared to control mice, the mRNA levels of TNF-α and IL-1β were significantly increased, but the mRNA levels of TGF-β and IL-10 were markedly decreased in CUMS mice. Moreover, the treatment of PB or IMI could reduce the expression of TNF-α and IL-1β but increase the expression of TGF-β and IL-10 (Fig. 5a-d). Interestingly, the trends of TNF-α, IL-1β, TGF-β and IL-10 concentrations in hippocampus of mice, which was determined by ELASA assay, were similar to the expression of them (Fig. 5e-h). These findings strongly proved that PB regulated inflammatory factors expression in CUMS mice.

### PB activated Nrf2/HO-1 signal pathway but inhibited the phosphorylation of NF-kB

**Fig. 6:**
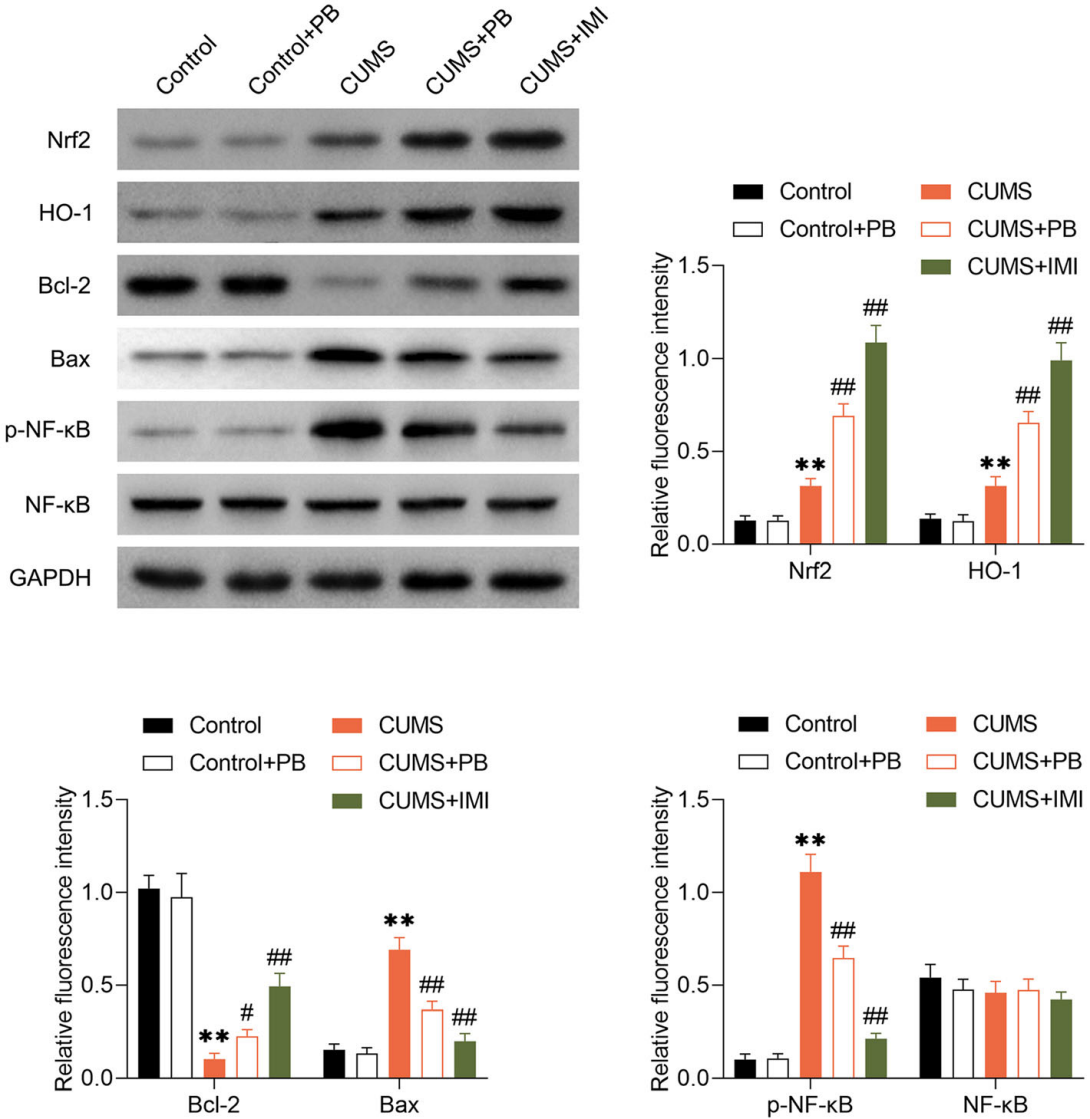
PB activated Nrf2/HO-1 signal pathway but inhibited the phosphorylation of NF-kB The changes of expression of Nrf2, HO-1, Bcl-2, Bax, p-NF-kB, NF-kB were assessed by western blot. **p < 0.01 vs. Control. #*p* < 0.05, ##*p* < 0.01 vs. CUMS

To further explore how PB ameliorated neuroinflammation and apoptosis, we observed the dynamic changes of Nrf2, HO-1 and p-NF-kB protein levels. From Fig. 6, the expression of HO-1 and Nrf2 were increased in CUMS mice. Nevertheless, the PB or IMI treatment could induce the Nrf2 and HO-1 expression, indicating that PB alleviated depressive-like behaviors in mice via activating Nrf2/HO-1 signal pathway. Compared to control mice, the Bcl-2 was decreased but the Bax was increased in CUMS mice, whereas these changes were reversed by PB or IMI treatment, suggesting that PB could inhibited the CUMS-induced cell apoptosis. More importantly, the phosphorylation of NF-kB was observed to promoted in CUMS mice but inhibited by PB or IMI treatment, indicating that PB alleviated depressive-like behaviors in mice via inhibiting the phosphorylation of NF-kB.

## Discussion

**Fig. 7:**
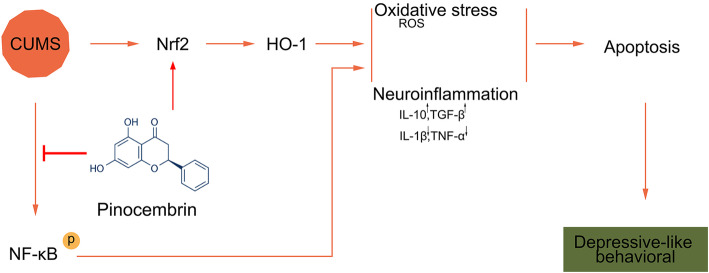
This cartoon depicted PB activated Nrf2/HO-1 signal pathway but inhibited the phosphorylation of NF-kB, and then ameliorated neuroinflammation and oxidative stress, further inhibited cell apoptosis and mitigated CUMS-induced depressive-like behaviors

We established a CUMS mouse model to mimic the depressive-like behaviors in vivo. PB alleviated the decreasing of sucrose preference and body weight. CUMS mice significantly increased the immobility time but decreased latency to abandon in FST, increased the immobility time in TST, and reduced crossing score and rearing score in OFT, whereas these changes were reversed by PB treatment. More importantly, PB decreased the concentration of ROS and MDA, but increased the SOD activity, suggesting that it could protected against oxidative stress in CUMS mice. Interestingly, PB inhibited cell apoptosis and regulated inflammatory factors expression in hippocampus of CUMS mice. Moreover, PB activated Nrf2/HO-1 signal pathway but inhibited the phosphorylation of NF-kB. Thus, PB activated Nrf2/HO-1 signal pathway but inhibited the phosphorylation of NF-kB, and then ameliorated neuroinflammation and oxidative stress, further inhibited cell apoptosis and mitigated CUMS-induced depressive-like behaviors (Fig. 7).

Depression caused mood and anxiety disorders and adversely affect individual’s productive life. Currently, natural products are considered good choices to treat 87% of all categorized human diseases, because of their anticancer, antibacterial, antiparasitic, anticoagulant, and immunosuppressant functions, such as PB (Newman et al. 2003). It has been reported that PB attenuated autonomic dysfunction and atrial fibrillation susceptibility (Ye et al. 2019). Gao J et.al have been pointed that PB inhibits the proliferation and migration but promotes the apoptosis in ovarian cancer cells (Gao et al. 2019). Moreover, PB has been proved to protect hemorrhagic brain primarily through inhibiting toll-like receptor 4 and decreasing M1 phenotype microglia (Lan et al. 2017). These results provided a novel strategy of PB for treating depression. In the present study, we have established a CUMS-induced depression-like behavior mouse model for in vivo study. Interestingly, we demonstrated that PB alleviated the decreasing of sucrose preference and body weight, and alleviated depressive-like behaviors in CUMS mice, indicating that PB may be a promising novel drug therapeutic for depression. However, the mechanism of how PB affects the depressive-like behaviors in CUMS-induced mice is still unclear.

Postmortem analysis show that the size of neurons is reduced and the apoptosis of neurons is increased in patients with depression (Stockmeier et al. 2004; McKernan et al. 2009). Stress induces cell apoptosis in the hippocampus and reduces neurogenesis in vivo (Kubera et al. 2011). In this study, compared to control mice, we have demonstrated that PB inhibited cell apoptosis in hippocampus of CUMS-induced mice. Oxidative stress causes mitochondrial membrane depolarization, leading to the release of cytochrome C from mitochondria into the cytoplasm, which activates the caspase pathway and leads to cell apoptosis (O'Rourke et al. 2005). Accumulating evidence indicate oxidative stress involves in the pathophysiologic hippocampus injury and plays a vital role in the CUMS-induced pathogenesis (Ahmad et al. 2010; Sakr et al. 2015; Chen et al. 2016; Jindal et al. 2013; Bhatt et al. 2014). The inflammatory stimuli are likely to be in response to apoptosis (Zonis et al. 2013; Zunszain et al. 2012). Fortunately, in current study, we found that PB inhibited the TNF-α and IL-1β expression, but increase the expression of TGF-β and IL-10, indicating that the PB might ameliorate neuroinflammation in CUMS-induced mice. Moreover, PB decreased the concentrations of ROS and MDA, but increased the SOD activity, suggesting that its protective function to alleviate oxidative stress. Thus, combined all these findings, we concluded that PB alleviate depressive-like behaviors via inhibiting oxidative stress and neuroinflammation, resulting in reducing cell apoptosis in CUMS-induced mice. Nevertheless, how PB influences the neuroinflammation and oxidative stress remains unclear.

There are many evidences highlight that PB serves as a target for Nrf2/HO-1 Axis, reduces autonomic dysfunction and susceptibility to atrial fibrillation via deactivating NF-κB/TNF-α pathway, inhibits the proliferation and migration but promotes the apoptosis through inhibiting N-cadherin and GABAB receptor (Ye et al. 2019; Gao et al. 2019; Habtemariam 2019). Interestingly, we confirmed that PB activated Nrf2/HO-1 signal pathway but inhibited the phosphorylation of NF-kB, which was partly consistent with previous studies. Nrf2-ARE pathway is proved to reduce oxidative stress and neuroinflammation, and play a protective role in neurodegenerative diseases (Buendia et al. 2016). Moreover, up-regulation of NF-kB perpetuates oxidative stress and contributes to neuroinflammation (Tobon-Velasco et al. 2014). However, how PB activates Nrf2/HO-1 signal pathway but inhibits the phosphorylation of NF-kB is still known, whether it could regulate some miRNAs or expression of proteins and then participate in Nrf2/HO-1 or NF-kB signal pathways? Hence, it still should pay more efforts for us to investigate the potential mechanism of PB in depression in clinic and animal models. Moreover, given that serum biomarkers relate to the degree of inflammation at brain site, we suspect that PB treatment might be associated with changes in peripheral inflammatory serum, which may provide a novel sight for explain the mechanism of PB in depression (Lattanzi et al. 2020; Di Napoli et al. 2018; Lattanzi et al. 2019).

## Conclusion

In conclusion, the present study demonstrated that PB reduces CUMS-induced hippocampal neuroinflammation and oxidative stress, thus inhibits cell apoptosis via activating Nrf2/HO-1 signal pathway but inhibited the phosphorylation of NF-kB. Our study proved an important role of PB against CUMS-induced neuroinflammation and apoptosis via Nrf2/HO-1 and NF-kB signaling pathway and provide a basis for investigating PB as a therapeutic strategy for the pathogenesis of depression.

## Data Availability

All data generated or analyzed during this study are included in this published article.

## Abbreviation

PB: Pinocembrin
CUMS: Chronic unpredictable mild stress
SPT: Sucrose preference test
OFT: Open field test
FST: Forced swim test
TST: Tail suspension test
ROS: Oxygen species
MDA: Malondialdehyde
SOD: Superoxide dismutase
IL: Inflammatory factor including interleukin
TNF: Tumor necrosis factor
TGF: Transforming growth factor
